# Pharmacological inhibition of CaMKK2 with the selective antagonist STO-609 regresses NAFLD

**DOI:** 10.1038/s41598-017-12139-3

**Published:** 2017-09-18

**Authors:** Brian York, Feng Li, Fumin Lin, Kathrina L. Marcelo, Jianqiang Mao, Adam Dean, Naomi Gonzales, David Gooden, Suman Maity, Cristian Coarfa, Nagireddy Putluri, Anthony R. Means

**Affiliations:** 1 Department of Molecular and Cellular Biology, Baylor College of Medicine, Houston, TX USA; 2 Adrienne Helis Malvin Medical Research Foundation, New Orleans, LA USA; 3 Department of Chemistry, Duke University, Durham, NC USA; 4 Dan L. Duncan Cancer Center, Baylor College of Medicine, Houston, TX USA; 5 Center for Drug Discovery, Baylor College of Medicine, Houston, TX USA

## Abstract

Binding of calcium to its intracellular receptor calmodulin (CaM) activates a family of Ca^2+^/CaM-dependent protein kinases. CaMKK2 (Ca^2+^/CaM-dependent protein kinase kinase 2) is a central member of this kinase family as it controls the actions of a CaMK cascade involving CaMKI, CaMKIV or AMPK. CaMKK2 controls insulin signaling, metabolic homeostasis, inflammation and cancer cell growth highlighting its potential as a therapeutic target for a variety of diseases. STO-609 is a selective, small molecule inhibitor of CaMKK2. Although STO-609 has been used extensively *in vitro* and in cells to characterize and define new mechanistic functions of CaMKK2, only a few studies have reported the *in vivo* use of STO-609. We synthesized functional STO-609 and assessed its pharmacological properties through *in vitro* (kinase assay), *ex vivo* (human liver microsomes) and *in vivo* (mouse) model systems. We describe the metabolic processing of STO-609, its toxicity, pharmacokinetics and bioavailability in a variety of mouse tissues. Utilizing these data, we show STO-609 treatment to inhibit CaMKK2 function confers protection against non-alcoholic fatty liver disease. These data provide a valuable resource by establishing criteria for use of STO-609 to inhibit the *in vivo* functions of CaMKK2 and demonstrate its utility for treating metabolically-related hepatic disease.

## Introduction

Calcium/calmodulin (Ca^2+^/CaM) is an essential complex that controls the activity of over 120 enzymes and proteins involved in numerous aspects of cell biology^1^, rendering inhibitors that directly target CaM unsuitable for *in vivo* studies^2^. Therefore, efforts have been aimed at developing small molecule antagonists to critical CaM targets in order to achieve beneficial therapeutic effects. A successful example of this strategy was the development of the immunosuppressive drugs cyclosporine and FK-506, which inhibit the activity of the only Ca^2+^/CaM-dependent protein phosphatase, calcineurin^3,4^. We recently opined that another such target was the Ca^2+^/CaM-dependent protein kinase kinase 2 (CaMKK2)^5,6^. CaMKK2 is an upstream initiator of a CaM kinase cascade as it activates a downstream pair of Ca^2+^/CaM-dependent proteins kinases, CaMKI and CaMKIV as well as the AMP-activated protein kinase (AMPK)^7,8^. Furthermore, CaMKK2 is significantly overexpressed in multiple tumor types and knockdown or inhibition of CaMKK2 reduced cell proliferation and tumorigenicity *in vivo* ^9,10^. In fact, the activity of CaMKK2 has emerged as an important regulator of metabolic and inflammatory processes that likely contribute to its impact on tumor growth^5,6^. Distinct from CaM, CaMKK2 displays cell type and tissue-specific expression, which creates a unique opportunity to develop pharmacological agents that selectively block its activity for treatment of a variety of pathologies^5,6^.

**Figure 1:**
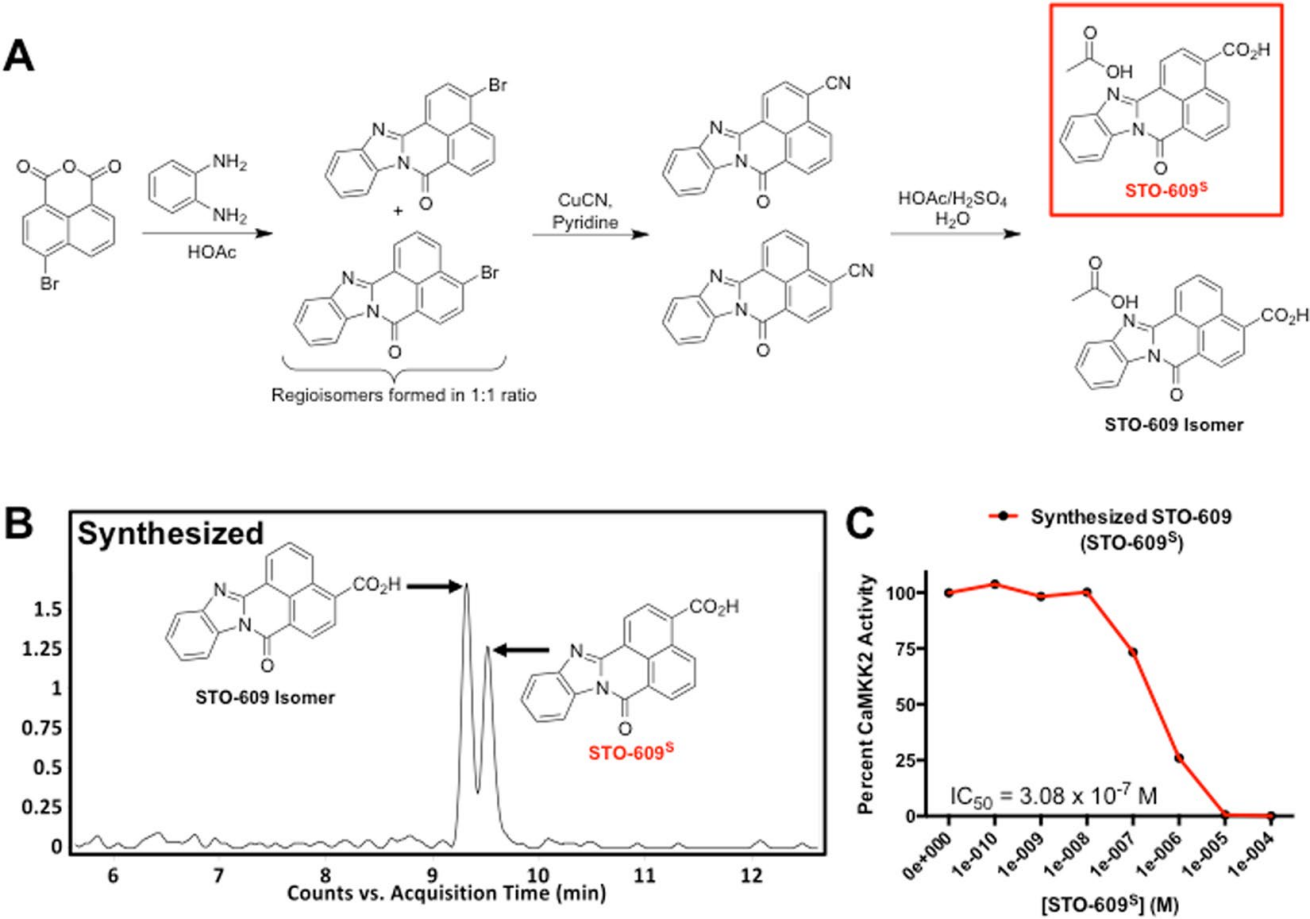
Synthesis and *In Vitro* Characterization of STO-609. (**A**) Schematic representation of the organic synthesis of STO-609. The chemical structure of synthesized STO-609 (STO-609^S^) is highlighted in the red box. **(B)** Chromatograms of STO-609^S^ showing identification of a unique chemical species with a *m/z* = 315 ([M + H]^+^. **(C)** Quantification of the efficacy STO-609^S^ for inhibition of CaMKK2 activity in a two-step kinase assay. Data are represented as percent CaMKK2 activity in the presence of increasing log doses of STO-609^S^. The accompanying inset lists the calculated IC_50_ value for STO-609^S^. Chemical structures were drawn using ChemBioDraw^48^.

At present, the most effective inhibitor of CaMKK2 activity is the ATP competitive inhibitor, STO-609 (7-Oxo-7*H*-benzimidazo[2,1-*a*]benz[de]isoquinoline-3-carboxylic acid acetate) (Fig. 1A, red box). Although few details exist regarding the history of STO-609, the proposed use of benzamidazoisoquinolines for the preparation of dyestuffs has been patented^11^. From a research perspective, published data describe STO-609 as a selective, cell-permeable inhibitor of both CaMKK1 and CaMKK2, although the K_*i*_ for CaMKK2 is more than 5 fold lower than that of CaMKK1^12^. A limited number of studies have reported *in vivo* use of STO-609 for inhibition of CaMKK2 in the control of satiety as well as to confer protection against prostate and liver cancers^9,13,14^. While these findings highlight the potential utility of STO-609 for attenuating downstream functions of CaMKK2 action *in vivo*, there have been no reports that evaluate the pharmacokinetics, tissue distribution, toxicity, dosing and metabolism of STO-609.

Herein, we present the first comprehensive analysis of STO-609 using a variety of *in vitro*, *ex vivo* and *in vivo* systems. Beginning with a batch synthesis of STO-609, we demonstrate the comparable efficacy of synthesized STO-609 (STO-609^S^) to that of commercial providers in a two-step *in vitro* kinase assay. Using recombinant human CYP450s and human liver microsomes, we identified CYP1A2 as the predominant P450 enzyme responsible for the metabolic conversion of STO-609 to three distinct mono-hydroxylated byproducts. Translating these observations to an *in vivo* setting using C57BL/6 J wild type mice, we characterized the toxicity, pharmacokinetics, tissue distribution and efficacy of STO-609 for inhibiting CaMKK2 function. Our findings identify the liver as the primary *in vivo* target of STO-609 when administered intraperitoneally, although potentially biologically relevant concentrations were also observed in the intestine, kidney, spleen and pancreas. Finally, published work from our laboratory has implicated CaMKK2 action in insulin resistance, perturbed hepatic metabolism and hepatocellular carcinoma^9,15^. Leveraging the information from our *in vivo* pharmacokinetic analysis of STO-609, we demonstrate that pharmacological inhibition of CaMKK2 reverses the hallmarks of hepatic steatosis in two mouse models of NAFLD. Taken together these data provide the research community with empirical information for the safe and effective dosing of mice with STO-609 and highlight the utility of pharmacological inhibition of CaMKK2 signaling to attenuate NAFLD.

## Materials and Methods

### Chemicals and Agents

Commercial STO-609 (STO-609^C^) (7-oxo-7H-benzo[de]benzo[4,5]imidazo[2,1-a]isoquinoline-3-carboxylic acid acetate) was purchased either from Tocris Bioscience (Bristol, U.K.) or Sigma-Aldrich (St. Louis, MO). α-Naphthoflavone, formic acid, and NADPH were obtained from Sigma-Aldrich (St. Louis, MO). Human liver microsomes (HLM), mouse liver microsomes (MLM) and the recombinant human CYP450s (EasyCYP Bactosomes) were purchased from XenoTech (Lenexa, KS). All the solvents for liquid chromatography-mass spectrometry (LC-MS) were of the highest grade from Honeywell - Burdick & Jackson (Muskegon, MI).

### STO-609 Synthesis

See Supplemental Fig. 1A for accompanying chemical structures associated with this portion of the synthesis of STO-609. A mixture of 4-bromo-1,8-naphthoic anhydride (25 g, 90.2 mmol) and *o*-phenylenediamine (12 g, 110 mmol, 1.2 eq.) in 17 M HOAc (500 ml) was heated to reflux for 4 hr. After this time, the reflux condenser was replaced with a short-path vacuum distillation head and the reaction mixture was concentrated to near dryness under reduced pressure. The suspension was transferred to a 4 L Erlenmeyer flask containing 1 L of ice water and a large magnetic stir bar. Solid NaHCO_3_ (~300 g) was added in small portions with stirring and the bright yellow insoluble material was removed by vacuum filtration. The filter cake was washed with saturated aqueous NaHCO_3_ (1 L), H_2_O (1 L) and dried *in vacuo* over the weekend giving the pure isomeric product (as determined by LC-MS, ESI-MS: (*m/z* = 349 ([M + H]^+^) as a bright yellow powder (31.2 g, 99%).

See Supplemental Fig. 1B for accompanying chemical structures associated with this portion of the synthesis of STO-609. The bromide (21 g, 60 mmol) was added portion-wise to pyridine (250 mL) in a 500 ml thick walled pressure vessel with stirring. Copper (I) cyanide (9.2 g, 102 mmol, 1.7 eq.) was then added in one portion and the mixture was heated in a heavy walled pressure vessel (150 °C, bath temperature) for 25 hr. The mixture was cooled to room temperature then poured into concentrated NH_4_OH (1000 ml)/ice (1000 ml). The resulting suspension was stirred until the ice melted. Insolubles were removed by vacuum filtration and the muddy brown filter cake was washed with H_2_O (1000 ml), 1 N HCl (1000 ml), H_2_O (1000 ml), then dried *in vacuo* yielding the crude product as a brown powder (22 g). This material was used in the final hydrolysis step without further purification.

See Supplemental Fig. 1C for accompanying chemical structures associated with this portion of the synthesis of STO-609. A mixture of the crude nitrile (21.1 g, 71 mmol), 17 M HOAc (200 ml), 18 M H_2_SO_4_ (80 ml) and H_2_O (60 ml) was heated to reflux for 24 hr after which time analysis of the reaction mixture by TLC (25% EtOAc in hexanes) indicated essentially complete consumption of starting nitrile. The reaction mixture was cooled to room temperature and diluted with ice H_2_O (1 L). The resulting brown suspension was stirred for 2 hr then subjected to centrifugation (2500 rpm, 5 min). The supernate was removed by decantation. The precipitate was washed with H_2_O (2 × 500 ml), and dried overnight *in vacuo*. The following morning the black-brown filter cake was suspended in 1 N NaOH (3 L). The resulting suspension was stirred at room temperature for 5 hr then filtered at the vacuum. The filtrate was extracted with methyl tert-butyl ether (6 × 2 L) then carefully acidified (pH 3–4) with 17 M HOAc. Insolubles were removed by filtration and dried *in vacuo* overnight. The resulting solid was suspended in MeOH (1 L) and filtered. The filtrate was concentrated to dryness under reduced pressure giving the product as a yellow-orange solid (15 g, 58%). ESI-MS: (*m/z* = 315 ([M + H]^+^). All chemical intermediates and final synthesized products were analyzed by Liquid Chromatography-Mass Spectrometry (LC-MS) and ^1^H/^13^C NMR. Synthesized STO-609 generated at the Duke Small Molecule Facility is at least 97% pure, which was determined by LC-MS analysis of the sample showing a single absorption peak at 5 different wavelengths. Area under those peaks provides the expected mass to charge ratio and ^1^H/^13^C NMR analysis of the sample showing the expected or reported resonances with no additional peaks. All additional analytical data for the compound are identical to commercial samples of STO-609.

### Preparation of STO-609 Dilutions

Synthesized STO-609 was dissolved in DMSO at 30 and 300 μM/kg and diluted in PBS prior to injection. Each concentration of STO-609 was prepared in a total volume of 2 ml, which was intraperitoneally injected into C57BL/6 J male mice as indicated. Rectal temperature was measured and blood collected at specified times after injection.

### Mice Studies

All animal experiments were performed under IACUC approved protocol number AN-6017 as reviewed by the Animal Care Research Committee at Baylor College of Medicine. All methods were performed in accordance with relevant guidelines and regulations. Adult male wild type C57BL/6 J mice (8–12 weeks old) were purchased from Jackson Laboratories either on normal chow or high fat diet as indicated. Mice were acclimatized for two weeks prior to experimentation and maintained on either normal chow or high fat diet as indicated. For all studies only male mice were used to avoid metabolic complications of unsynchronized estrous in female mice. Mice were maintained in a temperature controlled (23 °C) facility with a 12 hr light/dark cycle and fed 2920X Teklad Global rodent chow with free access to water. Two independent cohorts of mice were used for assessment of STO-609 to impact non-alcoholic fatty liver disease. One cohort of wild type C57BL/6 J male mice were subcutaneously injected at post-natal day 2 with streptozotocin (200 μg) followed by high fat diet feeding upon weaning at 3 weeks of age as previously described^16^. The second cohort of wild type C57BL/6 J male mice on high fat diet for 12 weeks was purchased from Jackson Laboratories. Each cohort was intraperitoneally injected with either DMSO (vehicle) or STO-609 (30 μM/kg) once per day for 4 weeks.

### *In Vitro* Kinase Assay

Assessment of commercial versus the batch synthesis of STO-609 for inhibition of CaMKK2 was performed as follows: kinase reactions were carried out in fresh HDMTC buffer (25 mM HEPES, 0.5 mM DTT, 10 mM MgCl_2_, 1 mM CaCl_2_, 0.1% Tween-20). Serial dilutions of commercial STO-609 (Tocris) or synthesized STO-609 (Duke Small Molecule Synthesis Facility) were prepared in DMSO. Bovine CaM was prepared as previously described^17^. MBP-AMPK^1–312^ was produced in *E. coli* and purified by HPLC as previously described^18^. GST-CaMKK2 was purchased from Sigma (C7370). SAMS peptide (HMRSAMSGLHLVKRR) was purchased from Abcam. Reactions containing final concentrations of CaM (1 μM), SAMS peptide (20μM), MBP-AMPK^1–312^ (0.4 μg/25 μl) and/or GST-CaMKK2 (4 nM) ± ATP (50 μM) and P^32^-γ-ATP (0.05 μCi/μl) were incubated in HDMTC buffer on ice in the presence or absence of increasing concentrations of STO-609. Reactions were incubated in a heat block at 30 °C for 20 min and stopped by the addition of 20 μl of the reaction mixture onto P81 Whatman filter paper. Filters were washed twice in a beaker containing 75 mM phosphoric acid (300 to 400 ml) for 20 min each. Filters were removed, air-dried, placed into individual scintillation tubes containing 3 ml of scintillation cocktail and radioactivity quantified using a Beckman LS 6500 scintillation counter.

### Metabolism of STO-609 in HLM and recombinant human CYP450s

Incubations of STO-609 with HLM or MLM were conducted in 1X phosphate-buffered saline (1X PBS, pH 7.4), containing 30 μM STO-609, 0.2 mg HLM or 2 pmol of each cDNA-expressed P450 enzymes (control, CYP1A2, 2A6, 2B6, 2C8, 2C9, 2C19, 2D6, 2E1, and CYP3A4) in a final volume of 190 μl as previously described^19^. After 5 min of pre-incubation at 37 °C, the reaction was initiated by addition of 10 μl of 20 mM NADPH (final concentration 1.0 mM) and continued for 30 min with gentle shaking. Incubations in the absence of NADPH were used as controls. Co-incubations of STO-609 (30 μM) and α-naphthaflavone (6.0 μM) in HLM were performed to confirm the role of CYP1A2 in STO-609 metabolism. Reactions were terminated by addition of 200 μl of ice-cold methanol, vortexing for 30 seconds and centrifugation at 15,000 × g for 15 min. Each supernate was transferred to an auto sampler vial and 5.0 μl was injected on to a UHPLC-QTOFMS system for analysis. Incubations were performed in duplicate for cDNA-expressed P450 enzyme and in triplicate for HLM inhibitory experiments.

### Sample Preparation for Pharmacokinetics and Tissue Distribution

Plasma samples were prepared by mixing 5.0 μl of plasma with 45 μl of ice-cold MeOH followed by centrifugation at 15,000 × g for 15 min to remove protein. The resulting supernates were transferred into autosampler vials for analysis. Tissues (liver, intestine, kidney, pancreas, spleen, lung, heart, testis, white adipose tissue, gastrocnemius and brain) were weighed and homogenized in H_2_O/MeOH (1:1 v/v, 100 mg tissue in 600 μl). A volume of 200 μl of MeOH was added to 100 μl of tissue extracts. After vortexing and centrifugation (15 min at 15,000 × g), each supernate was transferred to an autosampler vial and 5.0 μl was injected to a system combining ultra-high performance liquid chromatography (UHPLC) and quadrupole time of flight mass spectrometry (QTOFMS) for analysis. The concentration of STO-609 was calculated based on standard curves in the corresponding matrices. The pharmacokinetic parameters were calculated using non-compartmental analysis with PKSolver^20^. The linear trapezoidal rule was used for area under the curve (AUC) calculation.

### Measurement of Drug Metabolites

Separation of STO-609 and its metabolites was achieved using a LC-QTOFMS system (Agilent Technologies, Santa Clara, CA) equipped with a 100 mm × 2.1 mm (Agilent XDB C18) column as previously described^19^. Briefly, the column temperature was maintained at 45 °C. The flow rate was maintained at 0.3 ml/min with a gradient ranging from 2% to 98% aqueous acetonitrile containing 0.1% formic acid over a 15 min run. QTOFMS was operated in positive mode with electrospray ionization. Ultra high pure nitrogen was applied as the drying (12 L/min) and collision gas. The drying gas temperature was set at 325°C and nebulizer pressure was maintained at 35 psi. Capillary voltages were set at 3.5 kV. During mass spectrometry, real time mass correction and accurate mass were achieved by continuously measuring standard reference ions at *m/z* 121.0508, 922.0098 in the positive mode. The MS/MS of STO-609 and its metabolites was performed in targeted mode with a default isolation width of 4 *m/z* and collision energy ramp ranging from 20 to 50 V. Mass chromatograms and mass spectra were acquired by MassHunter® Workstation data Acquisition software (Agilent, Santa Clara, CA) in centroid and profile formats from *m/z* 100 to 1000. The acquisition rate was set as 1.5 spectra per second. Statistical analysis was conducted using Student’s independent *t*-test. Data are presented as mean ± s.e.m.

### Immunoblot Analysis

Immunoblot analyses were performed as described previously^21^. Briefly, proteins separated by SDS-PAGE, were transferred to nitrocellulose membranes, blocked in TBST (10 mM Tris-HCl (pH 8.0), 150 mM NaCl, 0.05% Tween-20) buffer supplemented with 5% bovine serum albumin (BSA) and incubated overnight with primary antibody in TBST with 5% BSA. Blots were incubated with an appropriate secondary antibody coupled to horseradish peroxidase, reacted with ECL reagents per the manufacturer’s (Amersham) suggestion and detected on X-ray film by autoradiography. The antibodies used are as follows: CaMKK2 (BD Transduction Laboratories: 610545), phospho-AMPK (Cell Signaling: 40H9), phospho-CaMKIVT196 (Santa Cruz: sc-28443), GAPDH (Cell Signaling: 14C10) and β-actin (Cell Signaling: 13E5).

### Gene Expression Profiling

RNA was isolated from liver using the RNeasy Mini Kit (Qiagen) according to the manufacturer’s protocol. First strand cDNA synthesis was performed using Superscript VILO according to the manufacturer’s instructions (Life Technologies, Carlsbad, CA). Real-time quantitative PCR was carried out using TaqMan Universal PCR Reaction Mix (Applied Life Technologies, Carlsbad, CA) with sequence-specific primers and the Mouse Universal Probe Library (Roche Applied Science, Indianapolis, IN). All gene expression assays were performed in technical duplicate and biological quintuplicate, and the resulting data normalized to endogenous *18S* rRNA expression. All qPCR data were analyzed using the ∆∆Ct method as previously described^22^. All primer sequences are available upon request.

### Sample Preparation for Tissue Metabolomics

Metabolites were extracted following the extraction procedure described previously^23–33^. Briefly, mouse tissues were stored at −80°C until analysis. For extraction of the metabolome, 25 mg of tissue was homogenized in 1:4 ice cold water:methanol mixture containing equimolar mixture of 11 standard compounds [Epibrassinolide, [D3] Testosterone (mass difference from endogenous Testosterone = 3 Da), [15 N] Anthranilic acid (mass difference from endogenous Anthranilic acid = 1 Da), Zeatine, Jasmonic acid, Gibberelic acid, [D4] Estrone (mass difference from endogenous Estrone = 4 Da), [15 N]- tryptophan (mass difference from endogenous Tryptophan = 1 Da), [D4] Thymine (mass difference from endogenous Thymine = 4 Da), [13 C] Creatinine (mass difference from endogenous Creatinine = 1 Da) and [15 N] Arginine (mass difference from endogenous Arginine = 1 Da)] as previously described^34^. This was followed by sequential addition of ice cold chloroform and water in 3:1 ratio and separation of the organic (methanol and chloroform) and aqueous solvents (water:methanol:chloroform:water; ratio 1:4:3:1). The aqueous extract was deproteinized using a 3 KDa molecular filter (Amicon Ultracel −3K Membrane, Millipore Corporation, Billerica, MA) and the filtrate containing metabolites was dried under vacuum (Genevac EZ-2plus, Gardiner, NY). Prior to mass spectrometry, the dried extract was resuspended in an identical volume of injection solvent composed of water:methanol (50:50) with appropriate solvent buffers and subjected to LC-MS.

### Targeted Metabolomics

Targeted metabolites were analyzed using a 6495 QQQ triple quadrupole mass spectrometer (Agilent Technologies) coupled to a 1290 series HPLC system via Selected Reaction Monitoring (SRM). Metabolites were measured using positive and negative ionization modes with an ESI voltage of + 3000 and −3500 ev, respectively. Approximately 9–12 data points were acquired per detected metabolite^28,35^.

### Glycolysis and TCA

Targeting glycolytic and TCA metabolites, the normal aqueous phase chromatographic separation was also used for targeted identification of metabolites as previously described^32^. Briefly, this method employed solvents containing water (solvent A), with solvent A modified by the addition of 5 mM ammonium acetate (pH 9.9) and 100% acetonitrile (ACN) (solvent B). The binary pump flow rate was 0.2 ml/min with a gradient spanning 80% B to 2% B over a 20 min period followed by 2% B to 80% B for a 5 min period and followed by 80% B for 13 min. The flow rate was gradually increased during the separation from 0.2 ml/min (0–20 mins), 0.3 ml/min (20.1–25 min), 0.35 ml/min (25–30 min), 0.4 ml/min (30–37.99 min) and finally set at 0.2 ml/min (5 min). Metabolites were separated on a Luna Amino (NH2) column (4 μm, 100 A 2.1 × 150 mm, Phenominex) maintained in a temperature-controlled chamber (37°C). All columns used in this study were washed and reconditioned after every 50 injections. Metabolites were measured using negative ionization mode with an ESI voltage of −3500 ev, respectively. Approximately 9–12 data points were acquired per detected metabolite.

### Separation of Amino acids, Carnitines and CoAs

Samples were delivered to the MS via normal phase chromatography using a 4.6 mm i.d × 10 cm Amide XBridge HILIC column (Waters) at 300 µl/min. Gradients were run starting from 85% buffer B (HPLC grade acetonitrile or 0.1% formic acid in acetonitrile) to 35% B from 0–3.5 min; 35% B to 2% B from 3.5–11.5 min; 2% B was held from 11.5–16.5 min; 2% B to 85% B from 16.5–17.5 min; 85% B was held for 7 min to re-equilibrate the column.

### Metabolomic Library

METLIN (Agilent Technologies) was used to search the unbiased mass spectral data. METLIN was created using approximately >2500 commercially available compounds whose retention times were defined using the Reverse Phase (RP) chromatographic method described above.

### Statistical Analyses

For unbiased methods (for each set of comparisons since the absence of some of the compounds in a group might have a biological connotation) the following conditions were used for initial selection of metabolite subsets. Regardless of the size of the biological cohort, metabolites must be observed in at least 50% of the samples to be considered as detected and kept for further analysis. Data were next median centered, log_2_ transformed, and then Inter Quartile Range (IQR) scaled. Following this procedure, a subset of metabolites was generated for any given set of comparisons treatments (i.e. DMSO vs. STO-609). This approach provided the input for the differential analyses involving non-parametric Mann-Whitney test before adjusting for multiple hypotheses correction using the Benjamini-Hochberg method. Significance was achieved for q-values < 0.25.

For the targeted metabolomics dataset, data were normalized using distinct internal standards for each metabolite collection, then log_2_ transformed for further processing. Specifically, TCA metabolites were normalized using L-Anthranallic acid, amino acid metabolites were normalized via L-Tryptophan, and CoAs were normalized based on L-Thymine. Differential metabolites were determined using a parametric two-sided t-test with unequal variance, adjusting for multiple hypotheses correction using the Benjamini-Hochberg method. Significance was achieved for q-values < 0.25.

For the targeted metabolomics dataset, based on the significantly differential metabolites for a specific comparison, we identified associated genes. Specifically, for a set of metabolites, the list of associated genes was obtained by querying the Human Metabolome Database (HMDB)^36^. For each gene list, pathway enrichment analysis was carried out using a hypergeometric distribution (significance at p-value < 0.05) against four different databases compiled in the Molecular Signatures Database (MSigDB)^37^: KEGG, Reactome, Gene Ontology Biological Processes and Hallmark. Based on these results, significantly enriched pathways, common between targeted datasets from both NAFLD models (STO-609 treatment over DMSO treatment) were selected. We reversed our analysis strategy for each selected pathway and comparison as follows: we first determined the genes enriched in the specific pathway, then their associated metabolites using HMDB, and finally via set union the subset of the significant metabolites associated with that pathway and comparison. The values for the pathway-associated metabolites were first averaged over the respective groups, then plotted in a pathway-centric manner in separate heatmaps.

### Data Availability

The datasets generated in the course of the current study are available from the corresponding author on reasonable request.

## Results

### Synthesis and *In Vitro* Characterization of STO-609

Since the amount of commercial STO-609 (STO-609^C^) required for comprehensive *in vivo* characterization is cost prohibitive, we performed a batch synthesis of STO-609 at the Duke Small Molecule Synthesis Facility. The synthetic schema used for our large-scale preparation of STO-609 yielded approximately 15 g of a yellow-orange solid (Fig. 1A, red box). Synthetic STO-609 (STO-609^S^) was verified by direct comparison of exact mass and retention time with those of commercial STO-609 (Tocris Bioscience) (Fig. 1B and Supplemental Fig. 1E).

A 2.4 Å crystal structure of the catalytic domain of CaMKK2 complexed with commercial STO-609 (Sigma Aldrich) has been previously reported and showed that the selective orientation of STO-609 binding to CaMKK2 is isomer specific^38^. It is important to note that this commercial STO-609 is an isomeric mixture as determined by MS analysis (Supplemental Fig. 1D). Consistent with these findings, comparison of commercial versus synthesized STO-609 using a two-step *in vitro* kinase assay showed nearly identical efficacy for inhibition of CaMKK2 activity considering the synthesized STO-609 (STO-609^S^) is approximately half as potent as the commercial due to the presence of the inactive isomer (Fig. 1C and Supplemental Fig. 1F). Collectively, these data suggest that our batch synthesis of STO-609 produced a mass spectrum consistent with that of commercial STO-609, and our synthetic product is efficacious for direct inhibition of CaMKK2 activity.

### Metabolic Analysis of STO-609 in HLM

**Figure 2:**
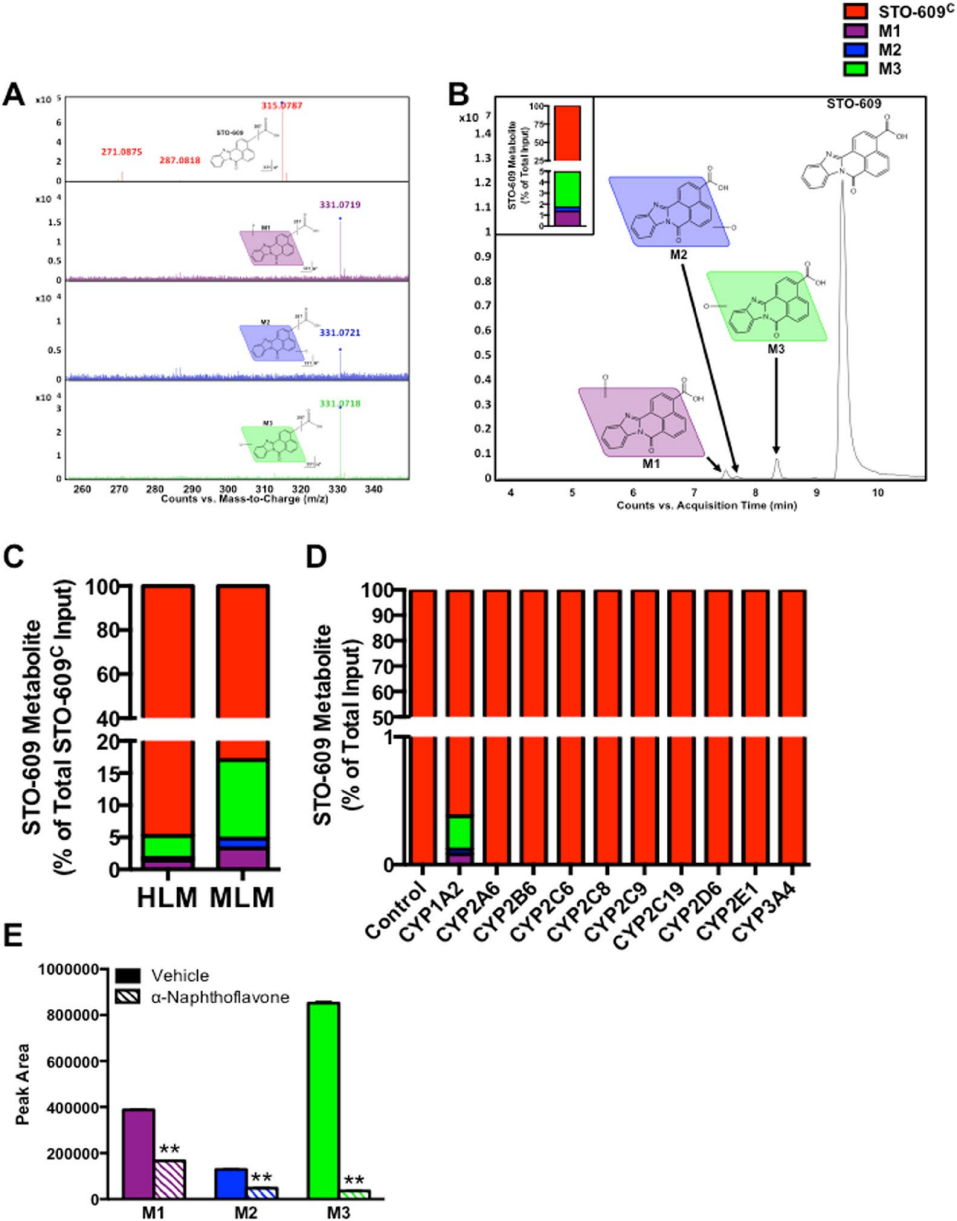
Metabolic Analysis of STO-609 in Human Liver Microsomes. (**A)** Representative MS/MS spectra of commercial STO-609 (STO-609^C^) metabolites M1 (purple), M2 (blue) and M3 (green) compared to that of STO-609^C^ (black). **(B)** Chromatograms of STO-609^C^ (red) and its metabolites (M1 (purple), M2 (blue) and M3 (green)) following incubation with human liver microsomes (HLM). Triplicate incubations were conducted in 1X PBS (pH 7.4) containing STO-609^C^ (30 µM), HLM (1.0 mg/ml) with or without NADPH (1.0 mM). The metabolites were quantified by UHPLC-QTOFMS. The accompanying inset shows the representative amount of each STO-609 metabolite calculated as a percent of total STO-609^C^. **(C)** Comparison of STO-609^C^ metabolism in human (HLM) versus mouse liver microsomes (MLM). The metabolites were quantified by UHPLC-QTOFMS. Data are graphed as the representative amount of each STO-609 metabolite calculated as a percent of total STO-609^C^. **(D)** cDNA-expressed P450s (CYP1A2, CYP2A6, CYP2B6, CYP2C8, CYP2C9, CYP2C19, CYP2D6, CYP2E1 and CYP3A4) were used to determine the role of individual CYP450 enzymes in the formation of STO-609 metabolites. All samples were analyzed by UHPLC-QTOFMS. Each STO-609 metabolite is represented as a percent of total STO-609^C^: M1 (purple), M2 (blue) and M3 (green). Conditions where no metabolites of STO-609 were identified are denoted as 100% of unmetabolized STO-609^C^ (red). **(E)** Validation of CYP1A2 in the formation of M1, M2 and M3 metabolites of STO-609^C^. Incubations were conducted in 1X PBS (pH 7.4) containing STO-609^C^ (30 µM), α-naphthoflavone (CYP1A2 inhibitor, 6.0 µM), HLM (1.0 mg/ml) and NADPH (1.0 mM). Metabolites were identified by UHPLC-QTOFMS. The peak area of the M1, M2 and M3 metabolites generated from the incubation of STO-609^C^ with HLM in the absence of α-naphthoflavone was set as 100%. All data are expressed as mean ± s.e.m. (n = 3). **P < 0.01. Chemical structures were drawn using ChemBioDraw^48^.

We next evaluated the metabolic processing of STO-609 using the well-defined human liver microsome (HLM) system. Incubation of commercial STO-609 (STO-609^C^) with HLM followed by MS analysis identified three mono-hydroxylated metabolic byproducts (Fig. 2A,B). The inset in Fig. 2B shows the relative abundance of these three mono-hydroxylated species of STO-609 upon incubation with HLM. We identified M3 as the most abundant metabolite of STO-609^C^ in HLM, followed by M1 and M2, respectively (Fig. 2B, inset). The putative structures of M1, M2 and M3 metabolites were identified based on their exact masses and MS/MS fragments (Fig. 2B). The metabolic processing of STO-609^C^ in HLM was compared to the analogous mouse liver microsome (MLM) system, which also identified three mono-hydroxylated species of STO-609^C^ (Fig. 2C). In both HLM and MLM systems, the majority of STO-609^C^ remained unmetabolized suggesting that primary metabolism may not represent the dominant mode of clearance for this compound.

Incubation of STO-609^C^ with cDNA-expressed P450s (control, CYP1A2, CYP2A6, CYP2B6, CYP2C8, CYP2C9, CYP2C19, CYP2D6, CYP2E1 and CYP3A4) revealed that CYP1A2 is the predominant enzyme responsible for STO-609 mono-hydroxylation (Fig. 2D). In fact, metabolism of STO-609^C^ by the isoenzyme CYP1A2 was sufficient to recapitulate the proportional abundance of the M1, M2 and M3 metabolites of STO-609^C^ produced upon incubation of STO-609^C^ with native HLM (compare Fig. 2B inset and Fig. 2D). CYP1A2 is selectively inhibited by the synthetic flavone, α-naphthoflavone^39^. Further confirming CYP1A2 as the predominant enzyme responsible for STO-609^C^ metabolism, the inhibitory effect on the production of M1, M2 and M3 was verified by co-incubation of α-naphthoflavone with HLM (Fig. 2E). The formation of M3 was suppressed > 90%, while the M1 and M2 metabolites of STO-609^C^ were attenuated approximately 50% by α-naphthoflavone (Fig. 2E). In summary, using individual recombinant P450 enzymes along with a selective CYP1A2 inhibitor identified that CYP1A2 functions to metabolize STO-609^C^ into three respective mono-hydroxylated species (Fig. 2E).

### *In Vivo* Analysis of STO-609 Toxicity and Pharmacokinetics

**Figure 3:**
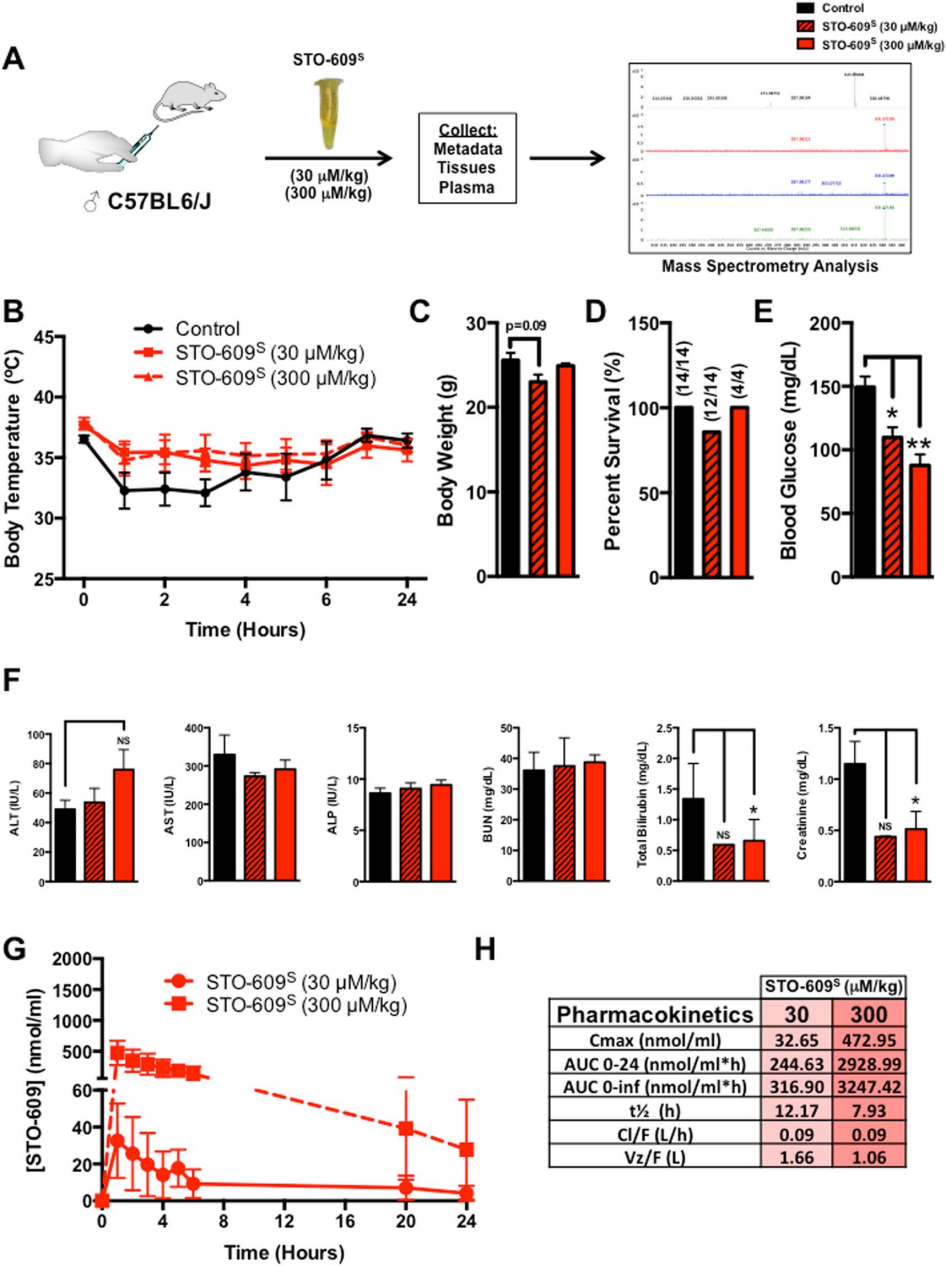
*In Vivo* Analysis of STO-609 Toxicity and Pharmacokinetics. (**A)** Schematic overview of the experimental approach for *in vivo* analysis of STO-609^S^ toxicity and pharmacokinetic properties. Adult male C57BL/6 J mice were i.p. injected with a single dose of STO-609^S^ (30 or 300 μM/kg). **(B)** Measurement of body temperature at various time points following i.p. injection with a single dose of STO-609^S^ (30 or 300 μM/kg). **(C)** Body weight measurements for cohorts of C57BL/6 J mice i.p. injected with a single dose of STO-609^S^ (30 and 300 μM/kg). **(D)** Survival plots for cohorts of C57BL/6 J mice i.p. injected with a single dose of STO-609^S^ (30 and 300 μM/kg). **(E)** Measurement of blood glucose levels in C57BL/6 J mice (N = 4 per dose) i.p. injected with a single dose of STO-609^S^ (30 and 300 μM/kg). **(F)** Measurement of plasma alanine aminotransferase (ALT), aspartate aminotransferase (AST), alkaline phosphatase (ALP), blood urea nitrogen (BUN), total bilirubin and creatinine levels in mice (N = 4 per cohort) injected with a single dose of STO-609^S^ (30 or 300 μM/kg). **(G)** STO-609^S^ plasma concentrations as determined by MS analysis in mice injected with a single dose of STO-609^S^ (30 or 300 μM/kg). Data are represented as the mean STO-609^S^ concentration for each time point ± s.e.m. **(H)** Pharmacokinetic parameters of STO-609^S^ in plasma. The parameters were calculated using PKSolver®. Definitions: C_max_, maximum (peak) plasma STO-609^S^ concentration; AUC_0–24_, area under the plasma STO-609^S^ concentration time curve between 0 and 24 h; AUC_0-INF_, area under the plasma STO-609 concentration time curve between 0 and infinity; t½, elimination half-life of STO-609^S^; Cl/F, apparent total clearance of STO-609^S^ from plasma; V_Z_/F, apparent volume of distribution of STO-609^S^ during the terminal phase after non-intravenous administration. Components of Fig. 3 were drawn using ChemBioDraw^48^.

To evaluate the *in vivo* toxicity of STO-609, C57BL/6 J male mice were intraperitoneally (i.p.) injected with a single dose of synthesized STO-609 (STO-609^S^) (30 or 300 μM/kg) (Fig. 3A). Body temperature (Fig. 3B), body weight (Fig. 3C), survival (Fig. 3D) and blood glucose (Fig. 3E) were monitored for 24 hr post-injection. Similar to mice administered the vehicle control (DMSO), all mice injected with STO-609^S^ showed an initial reduction in core body temperature regardless of the dose, although this effect was most pronounced in mice injected with vehicle (Fig. 3B). Injected mice were monitored for stress symptoms (i.e. body weight, ruffled fur, lethargy) for an additional 48 hr after the initial injection (Fig. 3C). Survival data reveal that a single intraperitoneal dose of STO-609^S^ failed to significantly impact survival (Fig. 3D). Recapitulating published data from our lab showing that *CaMKK2* ^−/−^ mice are more insulin sensitive^15^, STO-609^S^ treated mice display improved glycemia (Fig. 3E). To more comprehensively assess the effects of STO-609^S^ administration, plasma was collected immediately following the 24 hr time point from cohorts injected with each dose (30 and 300 μM/kg) and assayed for indices of hepatic and renal toxicity. Consistent with the marginal effects on body temperature and mortality, the low (30 μM/kg) and high doses (300 μM/kg) of STO-609^S^ did not induce parameters of liver (alanine aminotransferase (ALT), aspartate aminotransferase (AST) or alkaline phosphatase (ALP)) or kidney (blood urea nitrogen (BUN), bilirubin or creatinine) toxicity (Fig. 3F).

Following i.p. injection of STO-609^S^ (30 and 300 μM/kg), the concentration of STO-609 in plasma was measured by mass spectrometry to determine its *in vivo* pharmacokinetic properties. The concentration-time curves and pharmacokinetic properties of STO-609^S^ are summarized in Fig. 3G,H. Regardless of the dose, elimination of STO-609 from the plasma was consistent as indicated by the t½ ranging from ~8–12 hr (Fig. 3G,H). Determination of the C_max_ for each administered dose of STO-609^S^ showed tight correlation of dosing with the observed maximal serum concentration, suggesting no issues with absorption of STO-609^S^ in the circulation (Fig. 3G,H and Supplemental Fig. 2A). Clearance rates (Cl/F) were nearly identical for each dose of STO-609^S^ injected, which narrowly ranged from 0.06–0.09 L/hr (Fig. 3H). Calculation of the apparent volume of distribution (V_Z_/F) following i.p. administration of STO-609^S^ showed an inverse correlation with dose, which likely reflects the increasing volumes required to achieve higher doses of STO-609^S^ (Fig. 3H).

### Tissue Distribution of STO-609

**Figure 4:**
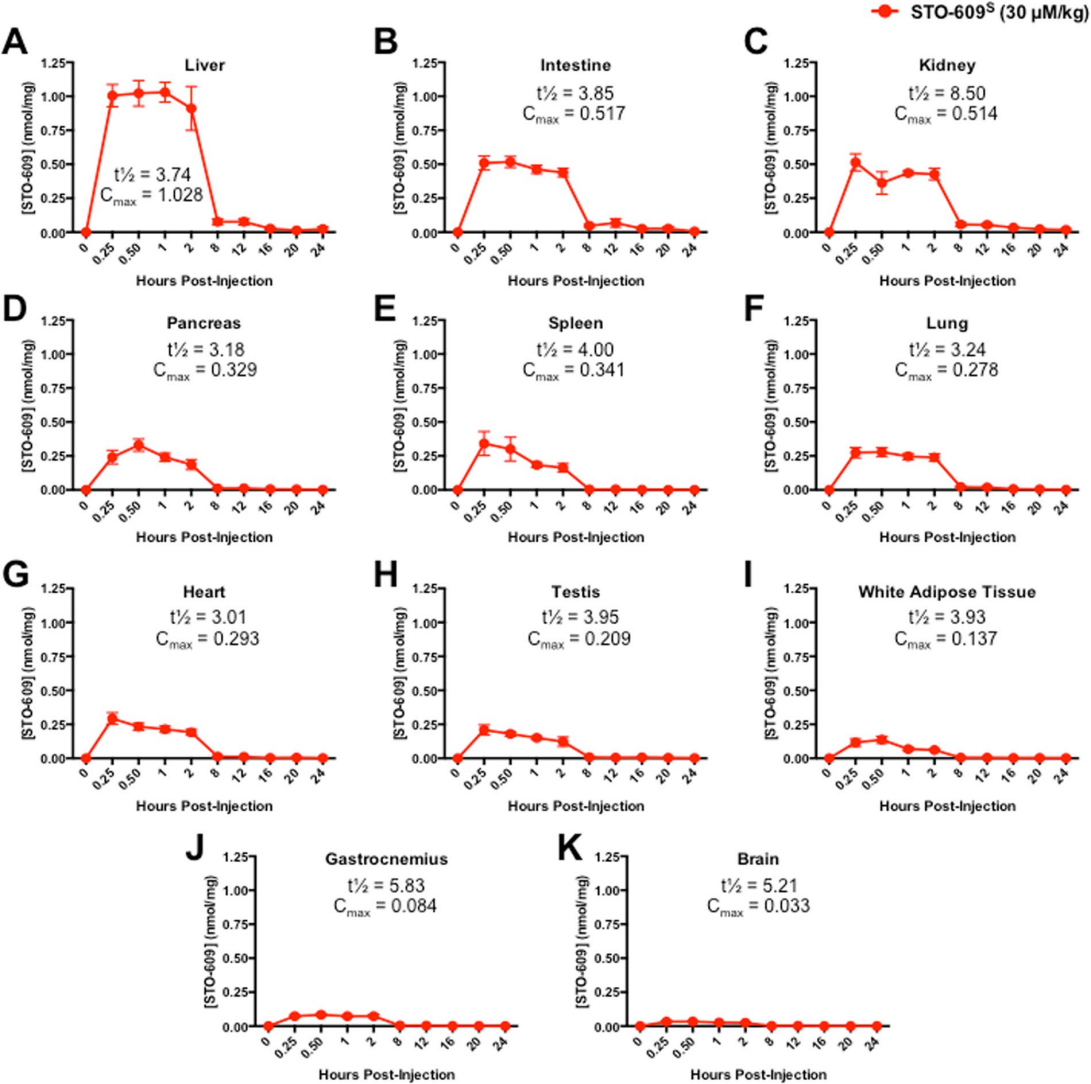
Tissue Distribution of STO-609. (**A)** STO-609^S^ concentrations in **(A)** liver, **(B)** intestine, **(C)** kidney, **(D)** pancreas, **(E)** spleen, **(F)** lung, **(G)** heart, **(H)** testis, **(I)** white adipose tissue, **(J)** gastrocnemius and **(K)** brain as determined by MS analysis in male C57BL6/J mice injected with a single dose of STO-609^S^ (30 μM/kg). Data are represented as the mean STO-609^S^ concentration for each time point ± s.e.m. Calculation of tissue specific t½ and C_max_ for STO-609^S^ accompanies each graph.

To determine the tissue-specific bioavailability of STO-609^S^, individual cohorts of C57BL/6 J male mice were intraperitoneally (i.p.) injected with a single dose of STO-609^S^ (30 μM/kg) and sacrificed at various time points post-injection (15 min, 30 min, 1, 2, 8, 12, 16, 20 and 24 hr). The concentration of STO-609^S^ was measured by mass spectrometry in target tissues that either express CaMKK2 or demonstrate CaMKK2-specific function (liver, intestine, kidney, pancreas, spleen, lung, heart, testis, white adipose tissue, skeletal muscle and brain)^5,6^ (Supplemental Fig. 3A). Not surprisingly, we identified the highest concentration of STO-609 in liver, which demonstrated a C_max_ of 1.028 nmol/ml and a t½ of 3.74 hr (Fig. 4A). Unlike our *in vitro* data on the metabolic processing of STO-609^S^ (Fig. 2), we found only the M1 metabolite of STO-609^S^ present in mouse liver tissue, albeit at very low concentrations compared to unmetabolized STO-609 (Supplemental Fig. 3B,C). Analysis of known CaMKK2 target genes^40^ by qPCR showed a STO-609^S^ dose-dependent decrease in *Ppargc1a* (*Pgc-1a*) and *G6pc* but no change in *Pepck* or *Ncoa1* (*Src-1*; negative control) (Supplemental Fig. 3D). Interestingly, we found a STO-609^S^ dose-dependent increase in *CaMKK2* gene expression (Supplemental Fig. 3D). Although this increase in *CaMKK2* gene expression was statistically insignificant, it mirrored an acute increase in CaMKK2 protein expression at the highest dose of STO-609^S^ (Supplemental Fig. 3E).

We also observed relatively similar amounts of STO-609 in the intestine and kidney, which exhibited a comparable C_max_ of ~0.5 nmol/ml yet distinct t½ of 3.85 for intestine and 8.5 hr for kidney (Fig. 4B,C). Likewise, we found that pancreas, spleen, lung, heart, testis and white adipose tissues displayed similar concentrations of STO-609 and exhibited comparable C_max_ and t½ values (Fig. 4D–I). Finally, we observed the lowest concentrations of STO-609 in skeletal muscle (gastrocnemius) and brain (Fig. 4J,K). These findings are partially expected, as the chemical nature of STO-609^S^ would likely preclude its ability to cross the blood brain barrier (BBB)^41^. Combined with the pharmacokinetic properties of STO-609^S^ observed in plasma (Fig. 3F–G), our tissue distribution data highlight the broad bioavailability of STO-609^S^ in a variety of peripheral tissues that have major implications for pathologies related to human disease.

### STO-609 Improves Hallmarks of NAFLD

**Figure 5:**
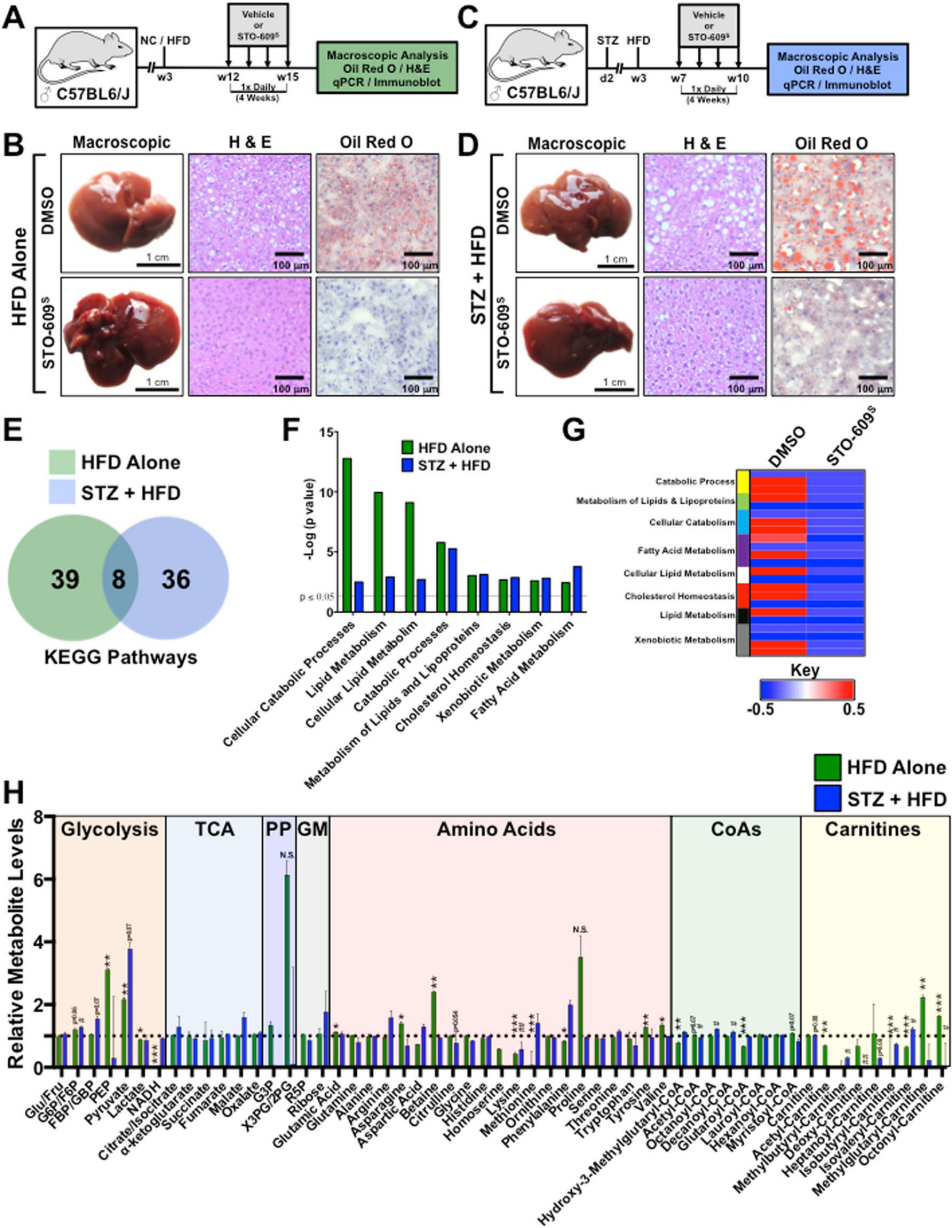
STO-609 treatment attenuates hallmarks of NAFLD. (**A)** Schematic overview of the experimental approach for *in vivo* assessment of STO-609^S^ on hepatic steatosis. Adult male C57BL/6 J mice were maintained on HFD containing 60% calories from fat immediately after weaning for the duration of experiment. After a 12-week exposure to HFD, mice were i.p. injected with either DMSO (vehicle) or STO-609^S^ (30 μM/kg) once per day for 4 weeks. Following the treatment period, mice were sacrificed and livers were isolated for downstream phenotypic and molecular analyses. **(B)** Representative macroscopic and histological analyses (H&E and Oil Red O staining) of C57BL/6 J mice maintained on HFD following a 4-week regimen of either DMSO (vehicle) or STO-609^S^ (30 μM/kg) treatment. **(C)** Schematic overview of the experimental approach for *in vivo* assessment of STO-609^S^ on a second model of hepatic steatosis. Male C57BL/6 J mice were subcutaneously injected at post-natal day 2 with low dose of streptozotocin (STZ) (200 μg) followed by high fat diet feeding containing 60% calories from fat immediately after weaning for the duration of experiment. After a 7-week exposure to HFD, mice were i.p. injected with either DMSO (vehicle) or STO-609^S^ (30 μM/kg) once per day for 4 weeks. Following the treatment period, mice were sacrificed and livers were isolated for downstream phenotypic and molecular analyses. **(D)** Representative macroscopic and histological analyses (H&E and Oil Red O staining) of STZ + HFD treated C57BL/6 J mice following a 4-week regimen of either DMSO (vehicle) or STO-609^S^ (30 μM/kg) treatment. **(E)** Targeted metabolomics were run on livers isolated from mice on either HFD alone or STZ + HFD models following the 4-week treatment with DMSO (vehicle) or STO-609^S^ (30 μM/kg). The resulting metabolomics data were used to generate gene association networks, which were subjected to KEGG pathway analysis. Both unique and overlapping KEGG pathways from HFD alone or STZ + HFD models are represented in Venn diagram format. **(F)** Shared KEGG gene ontology pathways between HFD alone and STZ + HFD models from (E) graphed as the –log (p value). **(G)** Heatmap representation of metabolites from shared ontological categories in HFD alone mice treated with DMSO (vehicle) or STO-609^S^ (30 μM/kg). **(H)** Quantitative metabolites representing glycolytic, TCA, pentose phosphate (PP) pathway, glycogen metabolism (GM), amino acid, acyl-CoAs and acyl-carnitines from HFD alone or STZ + HFD models. Metabolite levels for the DMSO treated groups were set to 1 and STO-609^S^ treated groups are shown relative to DMSO. Data are graphed as the mean ± s.e.m. For HFD alone: *P < 0.05; **P < 0.01; ***P < 0.001. For STZ + HFD: ^#^P < 0.05; ^#^P < 0.01. N.S. = not significant. Components of Fig. 5 were drawn using ChemBioDraw^48^.

Previously published work from our laboratory demonstrates that genetic ablation of *CaMKK2* is sufficient to protect mice against high fat diet-induced hepatic steatosis^15^. Leveraging the pharmacokinetic data of STO-609^S^ from plasma and liver (Fig. 3H and 4A), we tested the effect of STO-609^S^ in two mouse models of hepatic steatosis. In the first model, wild type C57BL/6 J male mice were maintained on HFD for 12 weeks to induce hepatic steatosis followed by daily injection of either DMSO (vehicle) or STO-609^S^ (30 μM/kg) for 4 weeks (Fig. 5A). Macroscopic and histological analyses show that STO-609^S^ treatment markedly improves hepatic steatosis as evidenced by H&E and Oil Red O staining (Fig. 5B). In a second murine model of NAFLD, wild type C57BL/6 J male mice were injected with streptozotocin (STZ) at postnatal day 2 and maintained on HFD for 7 weeks after weaning to induce hepatic steatosis as previously described^16^. Mice were injected daily with either DMSO (vehicle) or STO-609^S^ (30 μM/kg) for 4 weeks (Fig. 5C). Macroscopic and histological analyses show that STO-609 treatment markedly improves hepatic steatosis as evidenced by H&E and Oil Red O staining (Fig. 5D). Prolonged treatment with STO-609^S^ had no effect on body weight (Supplemental Fig. 4A,B), but showed a slight improvement in glycemia (Supplemental Fig. 4A,B), consistent with the acute effect on glycemia (Fig. 3E). Also, chronic exposure to daily STO-609 injections showed minimal impact on hepatic and renal toxicity in both NAFLD models (Supplemental Fig. 4C,D). We found that inhibition of CaMKK2 with STO-609^S^ differentially impacted known CaMKK2 substrates and target genes. In response to HFD alone, STO-609^S^ inhibition of CaMKK2 attenuated phosphorylation of AMPK^T172^ (Supplemental Fig. 4E) and decreased known target gene expression (Supplemental Fig. 4F). Conversely, in the STZ + HFD model, STO-609^S^ treatment failed to reduce phosphorylation of AMPK^T172^ or CaMKIV^T196^ (Supplemental Fig. 4G), yet showed reduction of known CaMKK2 target genes (i.e. *Pgc-1a* and *Pepck*) (Supplemental Fig. 4H).

To investigate how STO-609^S^ conferred improvement of hepatic steatosis, we performed targeted metabolomics on livers isolated from DMSO or STO-609^S^ treated mice from both NAFLD models. The resulting metabolite data from both NAFLD models (HFD alone vs. STZ + HFD) were used to assign ontological pathways impacted by STO-609^S^ treatment. Integration of these data revealed 8 common ontological pathways that were significantly influenced by STO-609^S^ treatment (Fig. 5E). Interestingly, we found that STO-609^S^ treatment suppressed metabolites associated with catabolic processes, including lipid and fatty acid metabolism (Fig. 5F,G). Most notably, mice treated with STO-609^S^ showed a concomitant increase in glycolytic metabolites and decreased acyl-CoAs and carnitines (Fig. 5H). The reduction in fatty acid species upon pharmacological inhibition of CaMKK2 suggests that blocking the activity of this enzyme either promotes β-oxidation or alternatively enhances the conversion and storage of these high-energy metabolites. Taken together, these findings clearly demonstrate attenuating CaMKK2 function with small molecule inhibitors is sufficient to not only inhibit, but reverse hallmarks of NAFLD in mice.

## Discussion

The data provided here establish the first comprehensive assessment of the metabolic processing, pharmacokinetics, toxicity and tissue bioavailability of STO-609 *in vivo*. Our findings demonstrate that STO-609^S^, when administered intraperitoneally, is well tolerated in mice with limited impact on renal or hepatotoxicity. Several studies using STO-609 in a variety of disease settings highlight its selectivity for inhibiting CaMKK2 activity. For example, work from our laboratory has established the importance of CaMKK2 in hypothalamic control of satiety through regulation of AMPK^13^. In fact, acute treatment of wild type, but not *CaMKK2* ^−/−^ mice, with STO-609 via cannulation of the third ventricle was sufficient to suppress appetite and promote weight loss. Similarly, recently published data emphasize the role of CaMKK2 in the onset and progression of hepatocellular carcinoma^9^. We showed through a variety of genetic studies that loss of *CaMKK2* blunts hepatic tumor cell growth, and treatment with STO-609 recapitulates these findings by regressing hepatic tumor burden *in vivo*. In other recent findings, genetic ablation of *CaMKK2* was shown to differentially impact liver metabolic homeostasis when mice were challenged with either low fat or high fat diets. More specifically, on low fat diet *CaMKK2* ^−/−^ mice show increased fat accumulation in the liver yet these mice display reduced hepatic steatosis on high fat diet^15^. These findings support the notion that CaMKK2 controls metabolic flexibility in the liver and are further supported by our observations here that STO-609^S^ treatment in two mouse models of NAFLD promotes reprogramming of glycolytic and fatty acid oxidation programs to reverse hepatic steatosis.

Also, our laboratory has reported that loss or inhibition of CaMKK2 activity confers protection against diet-induced obesity by improving pancreatic insulin secretion and increasing sensitivity of insulin-responsive tissues (i.e. liver and skeletal muscle)^15^. Directly in line with these published findings, our assessment of STO-609^S^ herein shows its potential for reducing circulating blood glucose levels in both normal chow and HFD fed mice. Given the broad tissue distribution of STO-609 *in vivo*, we anticipate that an extended regimen of STO-609 will be efficacious for improving whole body insulin sensitivity, especially in response to overnutrition^42^. This notion is partially supported by our observation that HFD fed mice treated daily with STO-609^S^ showed a reduction in blood glucose (Supplemental Fig. 4B). Due to the fact that genetic ablation of *CaMKK2* leads to a reduction in fat mass and an increase in lean muscle mass in mice fed a high fat diet^15^, it is conceivable that prolonged STO-609 treatment might improve these metabolic parameters in addition to its reported effects on enhancing insulin action^15^. The acute increase in body temperature resulting from STO-609^S^ treatment suggests it may act as a mitochondrial uncoupler, thus contributing to the positive effect on glucose tolerance and insulin sensitivity. Future studies will be required to address the potential impact of STO-609 treatment on energy expenditure and mitochondrial function. Although CaMKK2 expression is highest in various regions of the brain, we detected only minimal amounts of STO-609 in the brain suggesting its inability to traverse the BBB. While CaMKK2 function in the hypothalamus is important for controlling orexigenic gene expression to regulate satiety, the failure of STO-609 to adequately cross the BBB is perhaps advantageous, as other brain-centric functions of CaMKK2 such as mood and behavior are unlikely to be affected by STO-609 treatment^43^.

One important discovery generated from our work here was the identification of CYP1A2 as a P450 drug-metabolizing enzyme of STO-609. Although a relatively small portion of STO-609 was metabolized to three mono-hydroxylated species by CYP1A2, these data are intriguing in light of the reported ‘off-target’ effect of STO-609 on the aryl hydrocarbon receptor (AhR)^44^. Interestingly, STO-609 was shown to bind to and activate AhR, which in turn activates *CYP1A1* gene and subsequent protein expression of this P450 enzyme. Given our findings that the CYP1A family of cytochrome P450s is capable of metabolizing STO-609, we opine that the activation of AhR by STO-609 is not an off-target effect but rather a selective event that leads to induction of the appropriate drug metabolizing machinery required for partial STO-609 breakdown. Together, our data along with previously published work identifying α-naphthoflavone^45^ and furafylline^46^ as potent inhibitors of CYP1A2, create a potential combinatorial approach for extending the half-life and efficacy of STO-609 *in vivo*. Pharmacological inhibition of CYP1A2 with either α-naphthoflavone or furafylline would likely prohibit the metabolism of STO-609 *in vivo*. As our *ex vivo* or *in vivo* studies identified no additional metabolites of STO-609 degradation, presumably the aromatic mono-hydroxylation events catalyzed by CYP1A2 represent one mechanism by which STO-609 is inactivated and/or cleared. Perhaps the best-known example of such a combinatorial approach was reported in patients taking diazepam (valium), a benzodiazepine that acts as an allosteric modulator of GABA type A receptors used for treatment of anxiety or seizures, which requires the activity of CYP3A4 that is inhibited by drinking grapefruit juice^47^.

In its present form, the primary limitation prohibiting the effective *in vivo* use of STO-609 is its poor solubility. STO-609 is optimally soluble in either DMSO or NaOH, both of which complicate its use at high doses irrespective of the potential toxicity of STO-609 itself. The fact that the 2.4 Å crystal structure of the kinase domain of CaMKK2 complexed with STO-609 has been reported provides a molecular basis for designing improved derivatives of STO-609 that increase solubility while maintaining, or perhaps improving, its selectivity and efficacy for inhibition of CaMKK2, but not other related members of the CaMK family^38^. With knowledge from the solved crystal structure showing the orientation of STO-609 in the ATP binding pocket of the kinase domain of CaMKK2, molecular docking methods could be employed to design analogs of STO-609 that improve its solubility in biological media (i.e. 0.9% saline). Use of an *in silico* docking method could potentially identify regions of STO-609 most amenable for the addition of solubilizing groups to the molecular backbone while potentially minimizing effects on its binding affinity or specificity for CaMKK2. Although not comparatively tested in this study, it is possible that other modes of STO-609 delivery may prove more effective for preserving activity, extending the half-life or limiting the metabolic clearance of STO-609. Moving forward, it will be important to consider intravenous or oral delivery methods of STO-609. In addition to alternative delivery routes, our group is also interested in developing alternate formulations (i.e. nanoparticles, dextran derivatives, liposomes) for STO-609 to improve its bioavailability, tissue selectivity and half-life while concomitantly decreasing its toxicity.

The details provided in this study for the synthesis, activity, metabolism, pharmacokinetics, toxicity and tissue bioavailability of STO-609 for attenuating CaMKK2 activity *in vivo* provide a valuable resource for the research community. Taking advantage of these pharmacokinetic data, we provide the first application of this information by showing that a daily regimen of STO-609 is sufficient to regress hallmarks of hepatic steatosis. We anticipate that these findings will foster interest in the development of novel analogs and derivatives of STO-609 that, when combined with recent advances in drug delivery, will yield pharmacological agents with therapeutic efficacy against pathologies involving the actions of CaMKK2.

## Electronic supplementary material

Supplemental Figures and Legends
