# Supplementary material for: Pharmacological inhibition of CaMKK2 with the selective antagonist STO-609 regresses NAFLD
Source: Sci Rep. 2017 Sep 18;7:11793. doi: 10.1038/s41598-017-12139-3 (PMC5603587; doi:10.1038/s41598-017-12139-3)
Supplement: Supplementary file 1 — Supplemental Figures and Legends [file 41598_2017_12139_MOESM1_ESM.pdf]

Supplemental Figures and Figure Legends for:

Pharmacological inhibition of CaMKK2 with the selective antagonist STO-609 regresses NAFLD

Brian York[1,4,†], Feng Li[1], Fumin Lin[1], Kathrina L. Marcelo[1], Jianqiang Mao[1], Adam Dean[1], Naomi Gonzales[1,2], David Gooden[3], Suman Maity[1,4], Cristian Coarfa[1,4], Nagireddy Putluri[1] and Anthony R. Means[1,2,4]

[1] Department of Molecular and Cellular Biology, Baylor College of Medicine, Houston, TX

[2] Adrienne Helis Malvin Medical Research Foundation, New Orleans, LA

[3] Department of Chemistry, Duke University, Durham, NC

[4] Dan L. Duncan Cancer Center, Baylor College of Medicine, Houston, TX

Supplemental Figure 1

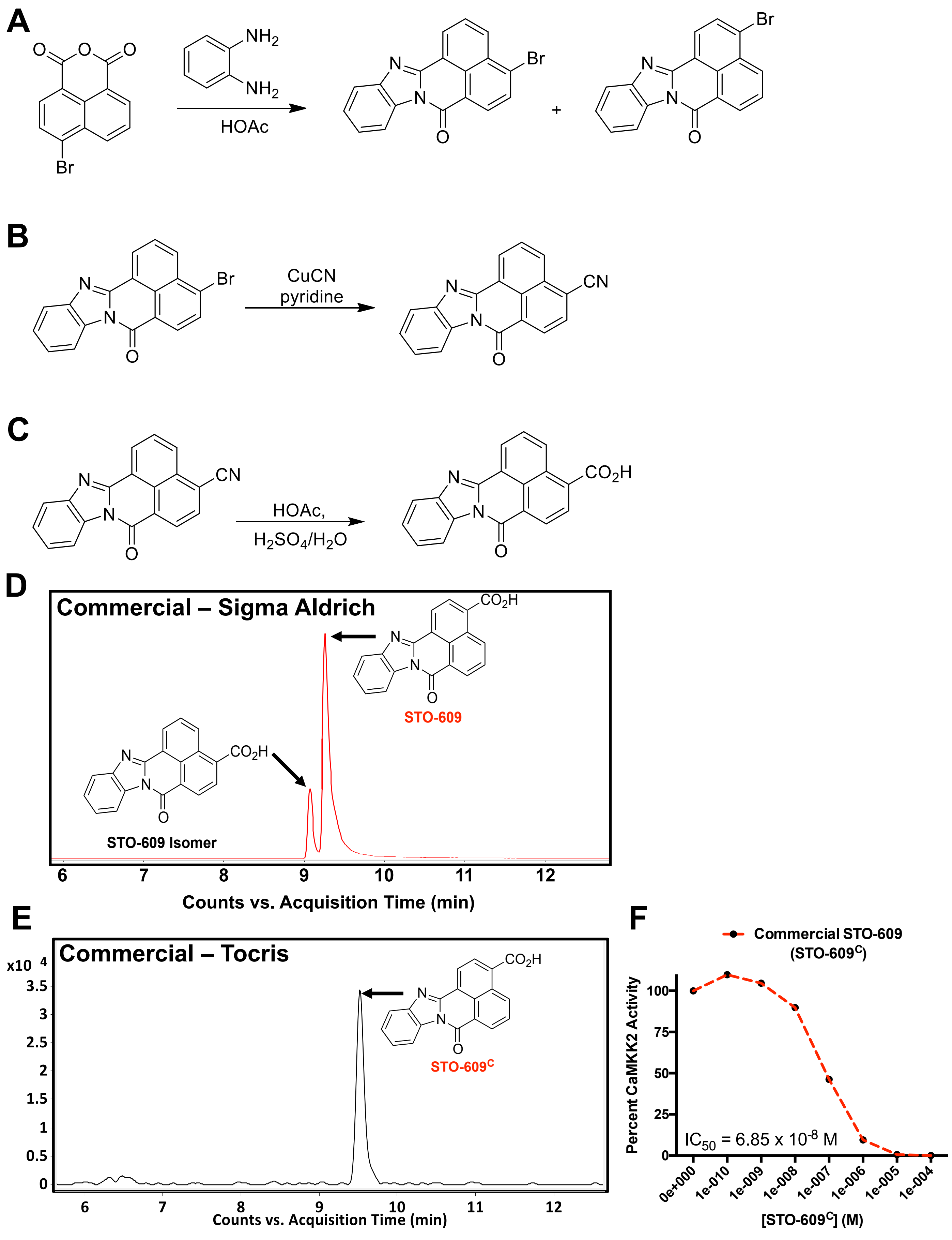

Supplemental Figure 1. Synthesis scheme for STO-609 and *In Vitro* Characterization of Commercial STO-609. (A) Chemical structures and workflow of the first step of STO-609 synthesis as described in the Materials and Methods. **(B)** Chemical structures and workflow of the first step of STO-609 synthesis as described in the Materials and Methods. **(C)** Chemical structures and workflow of the first step of STO-609 synthesis as described in the Materials and Methods. **(D)** Chromatogram of commercial STO-609 (Sigma Aldrich) showing identification of an isomeric mixture of STO-609 and its inactive isomer. **(E)** Chromatogram of commercial STO-609 (STO-609^{C}) showing identification of a unique chemical species with a *m/z* = 315 ([M+H]$^{+}$. **(F)** Quantification of the efficacy of commercial for inhibition of CaMKK2 activity in a two-step kinase assay. Data are represented as percent CaMKK2 activity in the presence of increasing log doses of STO-609^{C}. The accompanying inset lists the calculated IC_{50} value for STO-609^{C}. Chemical structures were drawn using ChemBioDraw[46].

Supplemental Figure 2

A

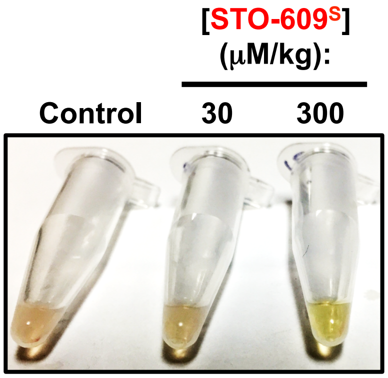

Supplemental Figure 2. ***In Vivo*** **Analysis of STO-609 Toxicity and Pharmacokinetics. (A)** Representative images of plasma samples isolated after 1 hr from mice i.p. injected with a single dose of DMSO (control) or STO-609^{S} (30 or 300 μM/kg).

Supplemental Figure 3

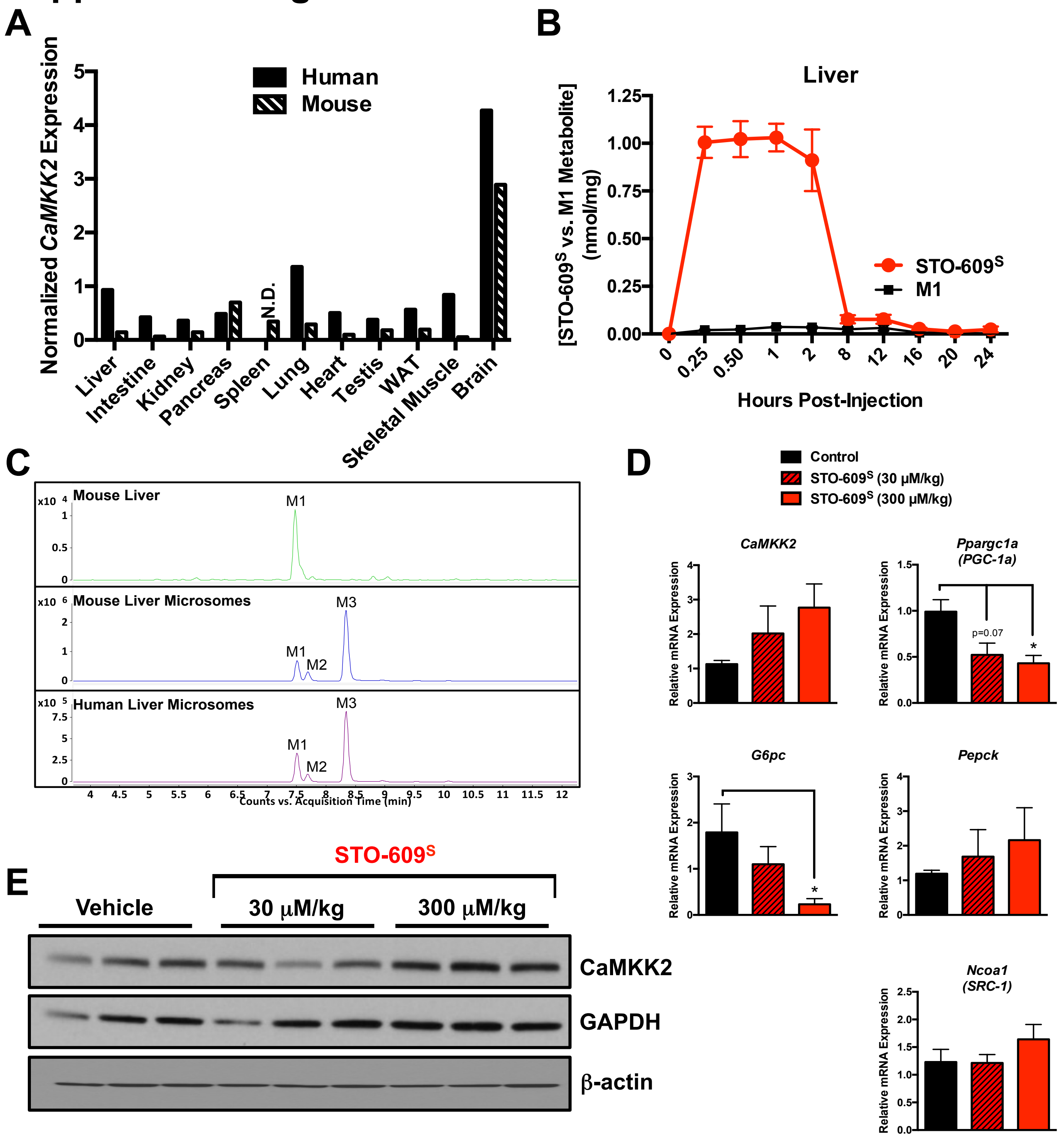

Supplemental Figure 3 - Continued

F

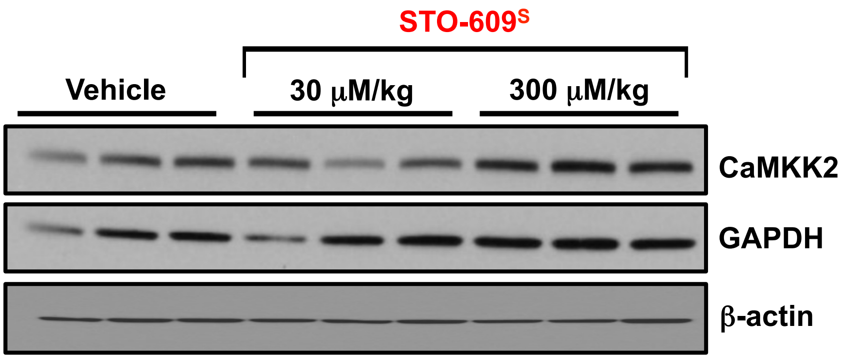

Original Data for Supplemental Figure 3E

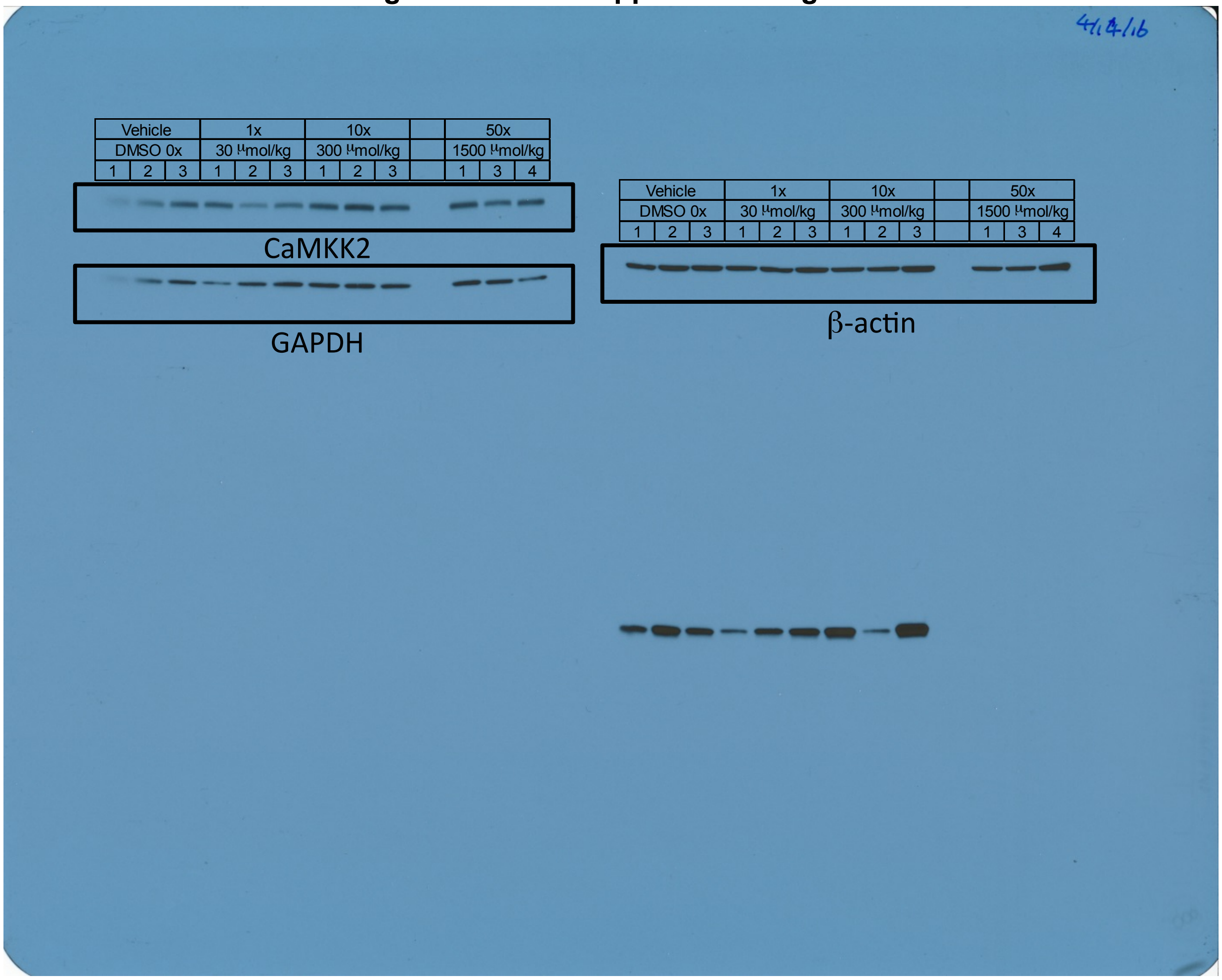

Supplemental Figure 3. Tissue mRNA expression of *CaMKK2* in human and mouse tissues. (A) Relative mRNA expression levels of *CaMKK2* in human and mouse tissues (liver, intestine, kidney, pancreas, spleen, lung, heart, testis, white adipose tissue (WAT), skeletal muscle and brain) that were investigated in this study for the bioavailability of STO-609^{S}. Data were graphed relative to the average mRNA *CaMKK2* levels determined across all tissues measured (www.biogps.org). **(B)** Concentration of the M1 STO-609^{S} metabolite (black line) compared to that of unmetabolized STO-609^{S} (red line) in liver as determined by MS analysis in mice injected with a single dose of STO-609^{S} (30 mM/kg). Data are represented as the mean STO-609^{S} or M1 metabolite concentration for each time point ± s.e.m. **(C)** Comparison of representative MS/MS spectra of STO-609^{S} metabolites in mouse liver (green), mouse (blue) and human (purple) liver microsomes. **(D)** qPCR analysis of *CaMKK2*, *Ppargc1a* (*Pgc-1a*), *G6pc*, *Pepck* and *Ncoa1* (*Src-1*) in liver tissue isolated from C57BL/6J mice (N=4 per dose) i.p. injected with either DMSO (control) or a single dose of STO-609^{S} (30 and 300 mM/kg) 24 hours after injection. **(E)** Immunoblot analysis of CaMKK2, GAPDH and b-actin (loading controls) in liver tissue isolated from C57BL/6J mice (N=3 per dose) i.p. injected with either DMSO (vehicle) or a single dose of STO-609^{S} (30 and 300 mM/kg). Images were cropped from the original immunoblots, which are included as supplementary information in Supplemental Figure 3. Data are graphed as the mean ± s.e.m. *$P < 0.05$. **(F)** Original immunoblot data for results shown in Supplemental Figure 3E.

Supplemental Figure 4

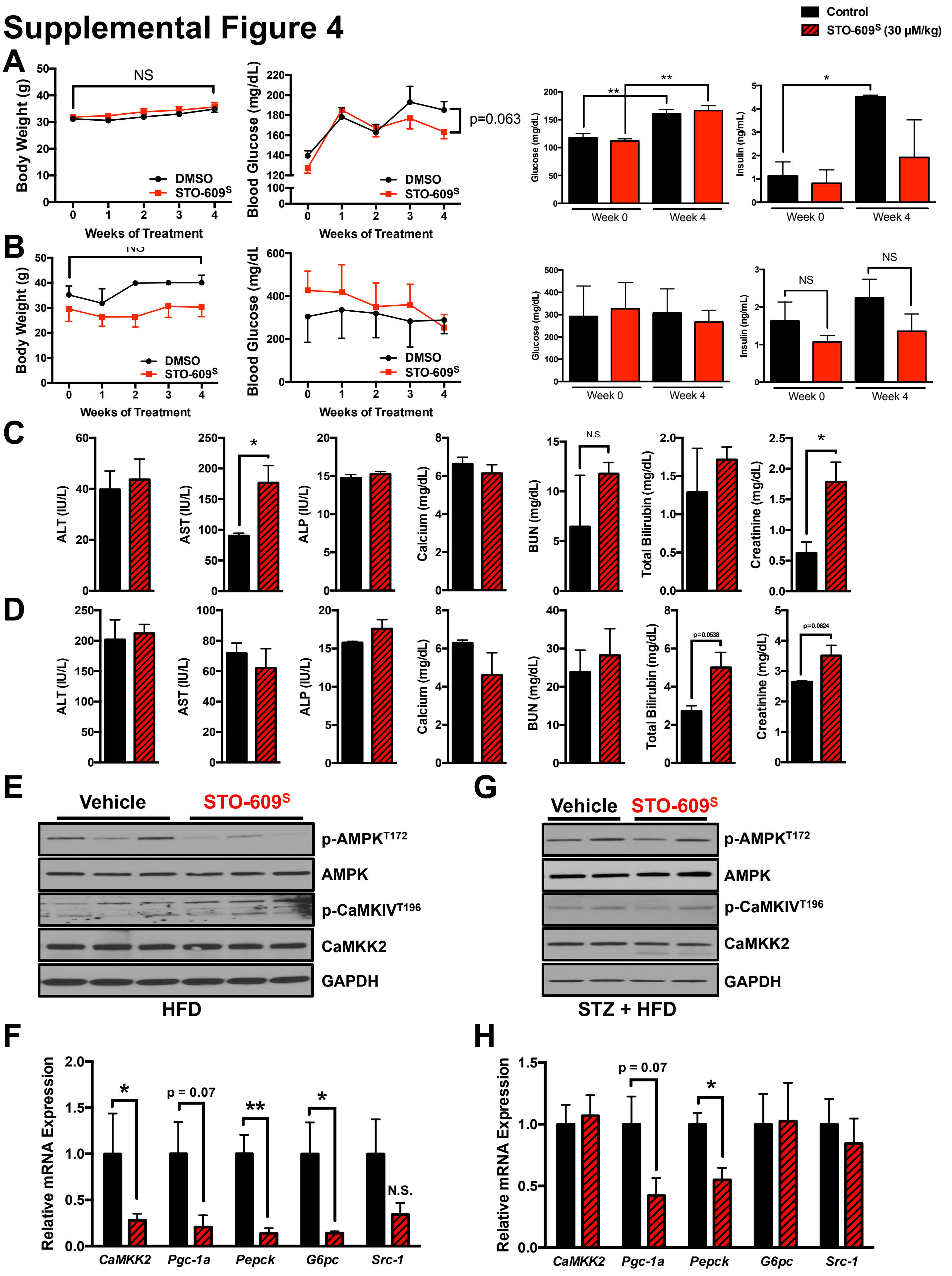

Supplemental Figure 4 - Continued

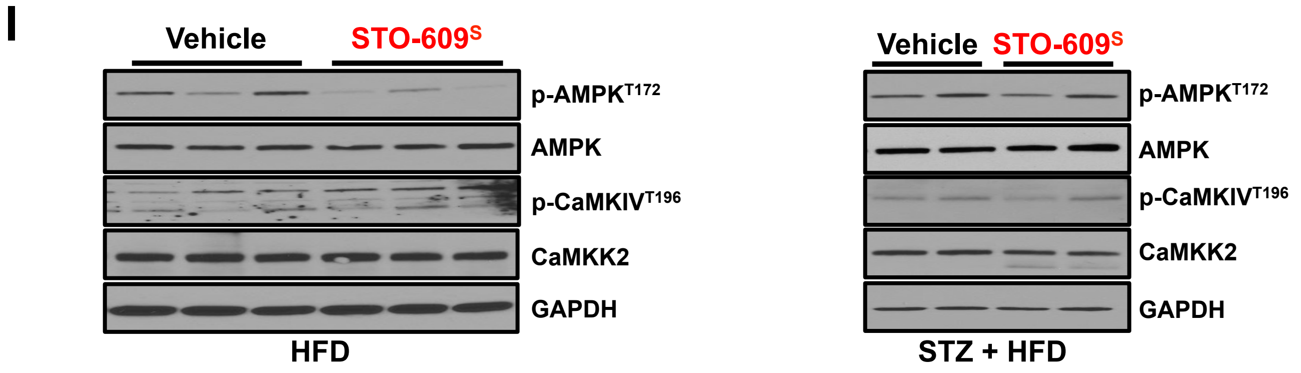

Original Data for Supplemental Figures 4F-G

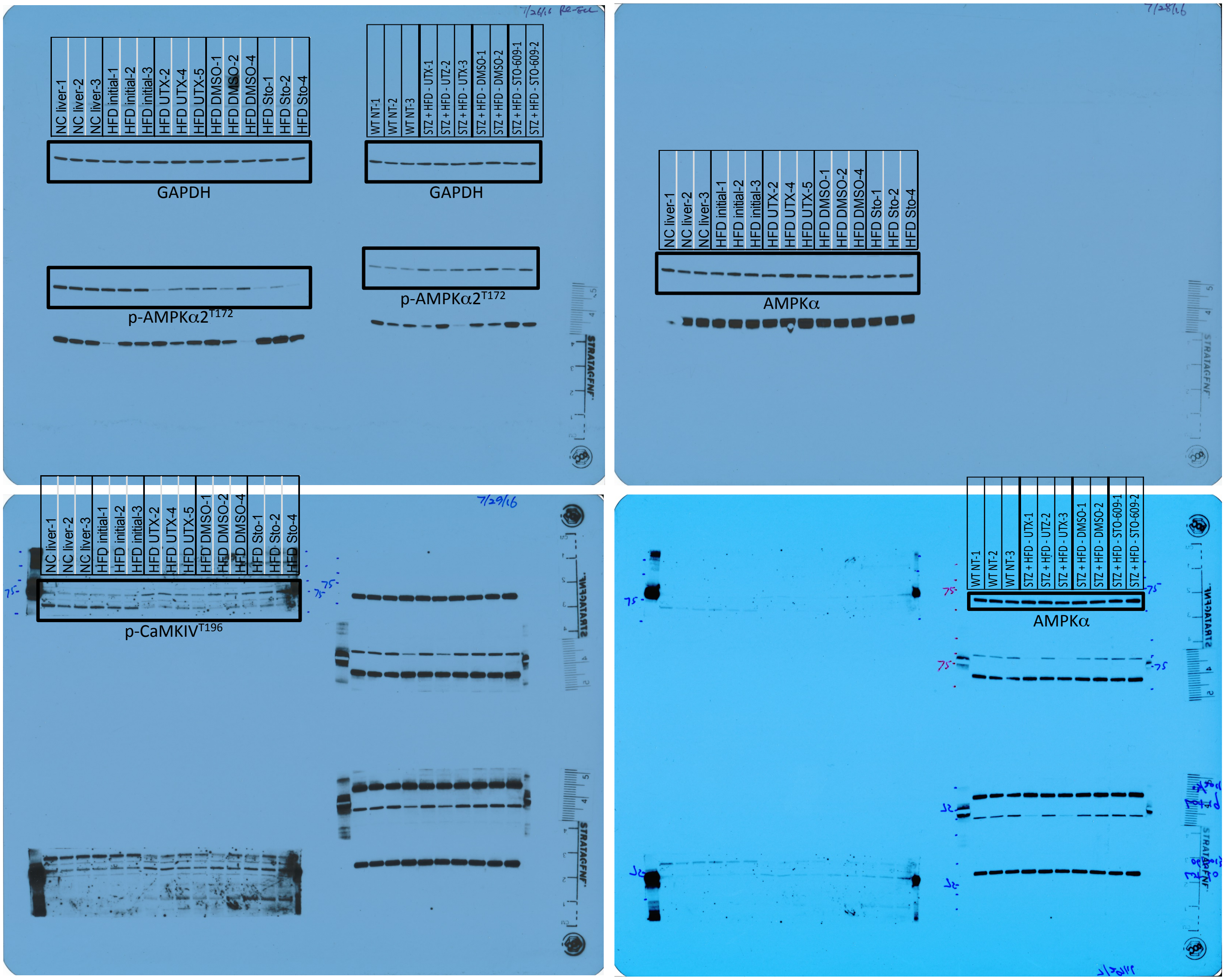

Supplemental Figure 4 - Continued

J

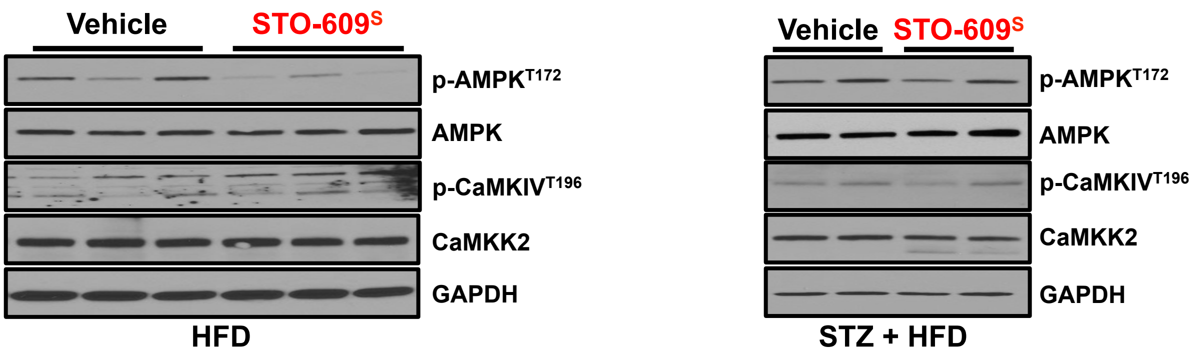

Original Data for Supplemental Figures 4F-G

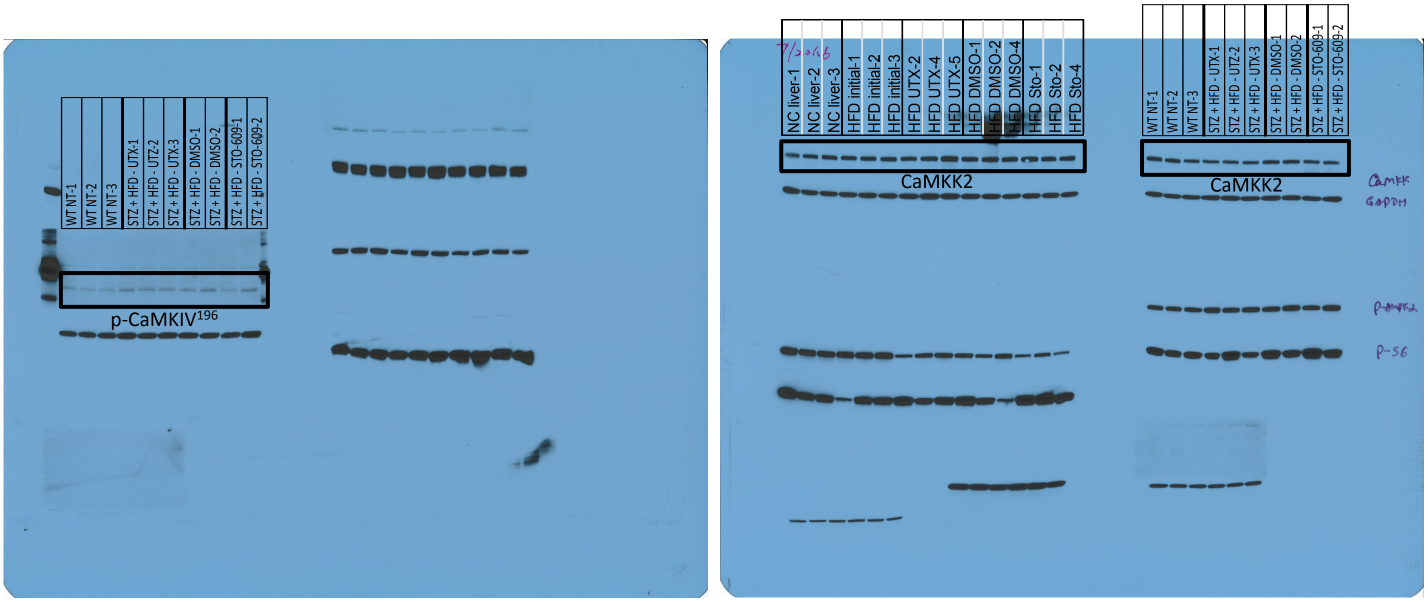

Supplemental Figure 4. Physiological and toxicity assessment of long-term STO-609 use. (A) Body weight, blood glucose and insulin measurements of HFD alone male C57BL6/J mice (N=3 per cohort) treated with daily injections of either DMSO (vehicle) or STO-609^{S} (30 μM/kg). **(B)** Body weight, blood glucose and insulin measurements of STZ + HFD male C57BL6/J mice (N=3 per cohort) treated with daily injections of either DMSO (vehicle) or STO-609^{S} (30 μM/kg). **(C-D)** Measurement of plasma alanine aminotransferase (ALT), aspartate aminotransferase (AST), alkaline phosphatase (ALP), calcium, blood urea nitrogen (BUN), bilirubin and creatinine levels in HFD alone (C) and STZ + HFD (D) mice (N=3 per cohort) following a 4-week daily regimen of DMSO or STO-609^{S} (30 μM/kg). **(E)** Immunoblot analysis of phospho-AMPK (T172), total AMPK, phospho-CaMKIV (T200), CaMKK2 and GAPDH (loading controls) in liver tissue isolated from HFD alone (N=3 per cohort) or STZ + HFD (N=2 per cohort) following a 4-week daily regimen of DMSO (vehicle) or STO-609^{S} (30 μM/kg). Images were cropped from the original immunoblots, which are included as supplementary information in Supplemental Figure 4. **(F-H)** qPCR analysis of *CaMKK2*, *Ppargc1a* (*Pgc-1a*), *Pepck*, *G6pc* and *Ncoa1* (*Src-1*) in liver tissue isolated from HFD alone (F) or STZ + HFD (H) mice (N=3 per cohort) following a 4-week daily regimen of DMSO (vehicle) or STO-609^{S} (30 μM/kg). Data are graphed as the mean ± s.e.m. *P < 0.05, **P < 0.01. **(I-J)** Original immunoblot data for results shown in Supplemental Figures 4E and 4G, respectively.
